# Performance Correction and Parameters Identification Considering Non-Uniform Electric Field in Cantilevered Piezoelectric Energy Harvesters

**DOI:** 10.3390/s24154943

**Published:** 2024-07-30

**Authors:** Xianfeng Wang, Hui Liu, Huadong Zheng, Guoxiong Liu, Dan Xu

**Affiliations:** 1 School of Civil Engineering and Architecture, Wuhan University of Technology, Wuhan 430070, China; xfwang91@whut.edu.cn (X.W.); drliuh@whut.edu.cn (H.L.); 2 Sanya Science and Education Innovation Park, Wuhan University of Technology, Sanya 572024, China; 3 China Railway 11th Bureau Group Corporation Limited, Wuhan 430064, China; zt11jlgx@163.com (G.L.); xudan.11g@crcc.cn (D.X.)

**Keywords:** piezoelectricity, energy harvesting, non-uniform electric field, performance correction, parameters identification

## Abstract

In the current electromechanical model of cantilevered piezoelectric energy harvesters, the assumption of uniform electric field strength within the piezoelectric layer is commonly made. This uniform electric field assumption seems reasonable since the piezoelectric layer looks like a parallel-plate capacitor. However, for a piezoelectric bender, the strain distribution along the thickness direction is not uniform, which means the internal electric field generated by the spontaneous polarization cannot be uniform. In the present study, a non-uniform electric field in the piezoelectric layer is resolved using electrostatic equilibrium equations. Based on these, the traditional distributed parameter electromechanical model is corrected and simplified to a practical single mode one. Compared with a traditional model adopting a uniform electric field, the bending stiffness term involved in the electromechanical governing equations is explicitly corrected. Through comparisons of predicted power output with two-dimensional finite element analysis, the results show that the present model can better predict the power output performance compared with the traditional model. It is found that the relative corrections to traditional model have nothing to do with the absolute dimensions of the harvesters, but only relate to three dimensionless parameters, i.e., the ratio of the elastic layer’s to the piezoelectric layer’s thickness; the ratio of the elastic modulus of the elastic layer to the piezoelectric layer; and the piezoelectric materials’ electromechanical coupling coefficient squared, ${k_{31}}^{2}$. It is also found that the upper-limit relative corrections are only related to ${k_{31}}^{2}$, i.e., the higher ${k_{31}}^{2}$ is, the larger the upper-limit relative corrections will be. For a PZT-5 unimorph harvester, the relative corrections of bending stiffness and corresponding resonant frequency are up to 17.8% and 8.5%, respectively. An inverse problem to identify the material parameters based on experimentally obtained power output performance is also investigated. The results show that the accuracy of material parameters identification is improved when considering a non-uniform electric field.

## 1. Introduction

Energy harvesting is indeed the process of capturing and converting ambient sources of energy, such as light, heat, vibration, or motion, into usable electrical energy. It is a promising technology used to develop self-sustaining systems so that the need for battery replacement and disposal can be minimized [1,2,3]. In settings devoid of prominent light or thermal sources, vibration energy harvesting emerges as a viable strategy [4,5,6,7,8,9]. For the purpose of harvesting vibration energy, piezoelectric materials serve as an optimal medium for converting vibration energy into electrical power, efficiently harnessing minor mechanical energy that is frequently overlooked or wasted [10,11,12,13,14,15,16,17,18,19,20,21,22]. Among virous piezoelectric transducers, the most commonly used structure in energy harvesting is the cantilevered beam or shaped cantilevered beam. A cantilevered piezoelectric energy harvester consists of a multilayer composite beam with one end secured to a host structure. In terms of layer arrangement, these harvesters primarily feature unimorph and bimorph configurations.

Numerous analytical models have been proposed to analyze the performance of cantilevered piezoelectric energy harvesters. Early efforts to model cantilevered piezoelectric energy harvesters drew inspiration from electromagnetic generator modeling [23,24], leading to the development of a lumped parameter model, which is often recognized as a single-degree-of-freedom model [25,26,27]. While the lumped parameter model initially provided valuable insight into the electromechanical coupling performance of cantilevered piezoelectric energy harvesters, it oversimplified by reducing the system to a single vibration mode of the bender, neglecting key aspects like modal shape and strain distribution. Building upon previous work, Sodano et al. [28] introduced enhanced modeling techniques for piezoelectric energy harvesters, employing approximate distributed parameter models based on Hamilton’s principle and the Rayleigh–Ritz method with Euler–Bernoulli beam assumptions. Following this path, Liao and Sodano further studied the optimization of cantilevered piezoelectric energy harvesters [29,30]. Shortly afterward, Erturk and Inman identified limitations in the above models [31,32]. Under Euler–Bernoulli beam assumptions, they then introduced fully coupled distributed parameter models with exact analytical solutions for these harvesters, incorporating both unimorph [33] and bimorph [34] configurations. Subsequently, considering Euler–Bernoulli, Rayleigh, and Timoshenko beam assumptions, Erturk developed a comprehensive framework for electromechanical modeling of piezoelectric energy harvesters utilizing the assumed-modes method [35].

In the aforementioned common modeling of cantilevered piezoelectric energy harvesters, the electric field strength is generally considered to be uniform, and it equals the output voltage divided by the piezoelectric layer thickness. This uniform electric field assumption seems reasonable at first glance, since the piezoelectric layer looks like a parallel-plate capacitor. However, for a piezoelectric bender, the strain distribution on a cross-section along the thickness direction is not uniform, which means the internal electric field generated by the spontaneous polarization should not be uniform. Following the electric field assumption of strongly coupled piezoelectric actuators [36], a non-uniform electric field is introduced to the modeling of strongly coupled piezoelectric bimorph harvesters by Gibus et al., resulting in a two-degree-of-freedom model [37]. Later, driven by the electric field distribution derived using the exact solutions for plane problems [38], Wang and Shi pointed out the critical issues induced by the uniform electric field assumption and proposed a non-uniform electric field in the piezoelectric layer [39]. In the research by Wang and Shi, an enhanced distributed parameter electromechanical model is developed, and the justification for incorporating non-uniform electric field consideration is systematically discussed.

With the introduction of a non-uniform electric field, improvements have been made to the conventional analysis models for piezoelectric energy harvesters. However, in current investigations, the performance correction arising from non-uniform electric field consideration is either neglected [37] or addressed only in specific instances involving certain sample harvesters with particular material parameters and geometric dimensions [39]. This means that the general determinants of performance correction and the influence of accounting for non-uniform electric fields on the identification of material parameters remain unclear.

In the present study, adopting non-uniform electric field assumption in the piezoelectric layer, a unified distributed parameter electromechanical model considering both unimorph and bimorph configurations is presented and further simplified to a more practical single-mode one. Based on this, the general determinates of performance correction induced by non-uniform electric field consideration are proposed regardless of specific material parameters and geometric dimensions. As for an inverse problem, the influence of accounting for non-uniform electric fields on the identification of material parameters is also investigated.

The rest of the paper is outlined as follows: In Section 2, a unified distributed parameter model for cantilevered piezoelectric energy harvesters is introduced, which contains unimorph and bimorph configurations as well as uniform and non-uniform electric filed assumptions. In Section 3, the non-uniform electric field in the piezoelectric layer is validated by comparing the power output performance predicted using the present model with that obtained using two-dimensional finite element analysis. In Section 4, using the unified model presented in Section 2, the determinants of performance correction induced by incorporating non-uniform electric field assumption are revealed. In Section 5, as an inverse problem, the influence of accounting for non-uniform electric fields on the identification of material parameters is investigated. And Section 6 concludes the study.

## 2. Unified Modeling of Cantilevered Piezoelectric Energy Harvesters

**Figure 1:**
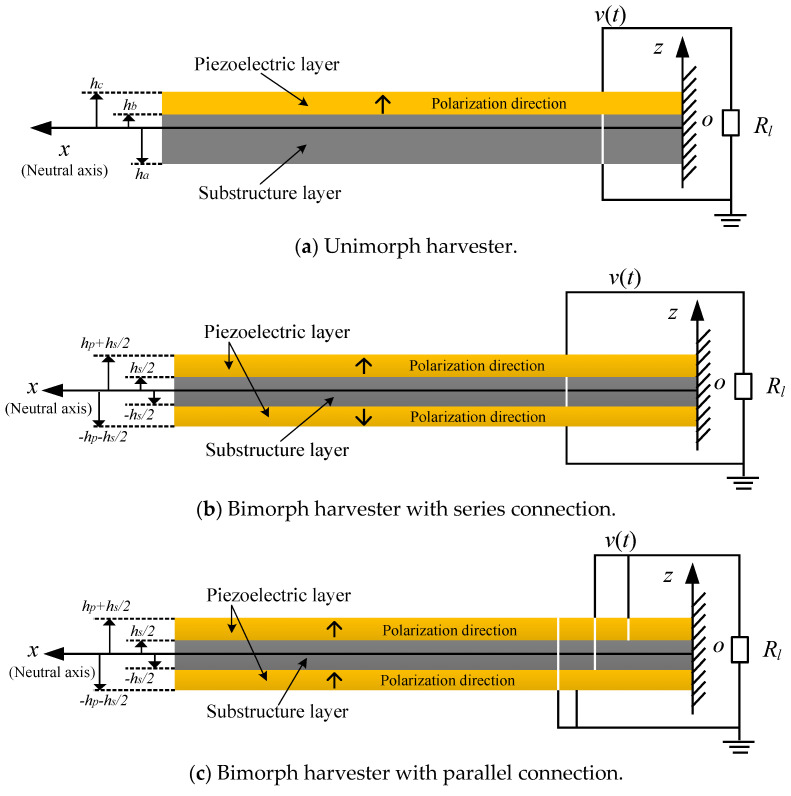
Typical configurations of cantilevered piezoelectric energy harvesters.

Cantilevered piezoelectric energy harvesters are primarily composed of three configurations: the unimorph harvester (Figure 1a), the bimorph harvester with series connection (Figure 1b), and the bimorph harvester with parallel connection (Figure 1c). These configurations can be conceptualized as a composite beam, incorporating elastic and piezoelectric layers arranged in various configurations.

### 2.1. Basic Equations

Considering the perspective of piezoelectricity, the constitutive equations for the elastic and the piezoelectric layers can be formulated as:

(1) $$T_{1}^{p}=Y_{p}\left(S_{1}^{p}-d_{31}E_{3}\right)$$

(2) $$D_{3}=d_{31}T_{1}^{p}+\varepsilon_{33}^{T}E_{3}$$

(3) $$T_{1}^{s}=Y_{s}S_{1}^{s}$$

Here, the subscript/superscript *p* and *s* indicate the piezoelectric and substructure layers, respectively. And the directions 1 and 3 align with the *x* and *z* directions. $T_{1}$ and $S_{1}$ denote stress and strain components along the *x* direction, respectively. *Y* represents the Young modulus, and $Y_{p}=1/s_{11}^{E}$, where $s_{11}^{E}$ represents the compliance coefficient of the piezoelectric material under a constant electric field. $d_{31}$ represents the piezoelectric constant, and $\varepsilon_{33}^{T}$ denotes the permittivity under constant stress. $E_{3}$ stands for the electric field, while $D_{3}$ stands for the electric displacement. In order to derive the one-dimensional material parameters $s_{11}^{E}$, $d_{31}$, and $\varepsilon_{33}^{T}$ from three-dimensional material parameters, it is necessary to employ a plane simplification approach and ensure zero stress along the 3-axis, as outlined in the Appendix A alongside the three-dimensional constitutive equations.

As an insulating material, the electric displacement must adhere to the electrostatic equilibrium equation, which can be expressed as:

(4) $$\frac{\partial D_{3}}{\partial z}=0$$

And the correlation between the potential distribution Φ and the electric field *E*_3_ is expressed as:

(5) $$E_{3}=-\frac{\partial \Phi }{\partial z}$$

The strain component in the harvester could be expressed in terms of the radius of curvature according to Euler–Bernoulli beam assumptions as follows:

(6) $$S_{1}\left(x,z,t\right)=-\frac{z\partial^{2}w_{rel}\left(x,t\right)}{\partial x^{2}}$$

Here, *z* denotes the position from the neutral axis, and $w_{rel}(x,t)$ represents the relative transverse deflection of the beam. When a harvester experiences a base motion, that reflected on the beam can be described as:

(7) $$w_{b}\left(x,t\right)=g\left(t\right)+xh\left(t\right)$$

where g(t) and h(t) are a traverse motion and a slight rotation at the base. Neglecting the extra excitation caused by the air, the mechanical governing equation of a cantilevered beam could be formulated as:

(8) $$\frac{\partial^{2}M\left(x,t\right)}{\partial x^{2}}+c_{s}I\frac{\partial^{5}w_{rel}\left(x,t\right)}{\partial x^{4}\partial t}+c_{a}\frac{\partial w_{rel}\left(x,t\right)}{\partial t}+m\frac{\partial^{2}w_{rel}\left(x,t\right)}{\partial t^{2}}=-m\frac{\partial^{2}w_{b}\left(x,t\right)}{\partial t^{2}}$$

In this expression, M(x,t) denotes the internal bending moment. $w_{rel}(x,t)$ and *m* represent the relative transverse deflection and the mass per unit length of the beam, respectively. $c_{s}I$ represents the equivalent damping term arising from structural viscoelasticity (where $c_{s}$ denotes the equivalent coefficient of strain rate damping and *I* is the equivalent area moment of the cross section), $c_{a}$ is the coefficient of viscous air damping, and these two distinct damping factors are assumed to account for the strain rate damping and viscous air damping, respectively. A comprehensive discussion on addressing the damping mechanism in cantilevered harvesters can be found in reference [32].

The electrical governing equation of a composite piezoelectric beam subjected to base motion connected to an external resistance $R_{l}$ could be expressed as:

(9) $$\frac{dq\left(t\right)}{dt}=\frac{v\left(t\right)}{R_{l}}$$

where v(t) denotes the output voltage and q(t) represents the free charge generated by the energy harvester, which could be characterized by:

(10) $$q\left(t\right)=\int_{A}D\cdot n\;dA$$

Here, **D** represents the electric displacement vector on the surface of the piezoelectric layer, while **n** is the unit normal vector to the electrode.

### 2.2. Non-Uniform Electric Field in Cantilevered Piezoelectric Energy Harvesters

In traditional modeling of cantilevered piezoelectric energy harvesters, the electric field in the piezoelectric layer is generally considered to be uniform as the ratio of the output voltage to the thickness of the piezoelectric layer. However, if the electric displacement $D_{3}$ is calculated by substituting a uniform electric field $E_{3}$ and Equation (6) into Equation (2), the obtained electric displacement obviously does not adhere to the electrostatic equilibrium equation, i.e., Equation (4). Since the divergence of electric displacement signifies the density of free charges, the electrostatic equilibrium equation inherently implies that the free charge within an insulator must equate to zero. Consequently, within the Euler–Bernoulli beam framework, the assumption of a uniform electric field distribution contradicts a fundamental physical premise, namely, the absence of free charges within an insulator.

Herein, we combine Equations (1), (2), (4) and (6). A newly proposed non-uniform electric field can be resolved as:

(11) $$E_{3}\left(x,z,t\right)=\frac{d_{31}Y_{p}}{\varepsilon_{33}^{S}}\frac{\partial^{2}w_{rel}\left(x,t\right)}{\partial x^{2}}z+B\left(x,t\right)$$

where $\varepsilon_{33}^{S}=\varepsilon_{33}^{T}-{d_{31}}^{2}Y_{p}$ is the permittivity at constant strain The corresponding electric potential could be resolved as:

(12) $$\Phi \left(x,z,t\right)=-\frac{d_{31}Y_{p}}{\varepsilon_{33}^{S}}\frac{\partial^{2}w_{rel}\left(x,t\right)}{\partial x^{2}}\frac{z^{2}}{2}-B\left(x,t\right)z+C\left(x,t\right)$$

where $B\left(x,t\right)$ and $C\left(x,t\right)$ are integration constants that can be solved using the potential boundary conditions at the surface of the piezoelectric layer, as illustrated in Figure 1. Specifically, for unimorph harvesters, the electric field and electric potential can be solved as:

(13) $$\left\{ \begin{array}{l} E_{3}\left(x,z,t\right)=\frac{d_{31}Y_{p}}{\varepsilon_{33}^{S}}\frac{\partial^{2}w_{rel}\left(x,t\right)}{\partial x^{2}}\left(z-h_{pc}\right)-\frac{v\left(t\right)}{h_{p}}\\ \Phi \left(x,z,t\right)=\frac{d_{31}Y_{p}}{\varepsilon_{33}^{S}}\frac{\partial^{2}w_{rel}\left(x,t\right)}{\partial x^{2}}\left(-\frac{z^{2}}{2}+h_{pc}z-\frac{h_{b}h_{c}}{2}\right)+\frac{v\left(t\right)}{h_{p}}\left(z-h_{b}\right) \end{array}\right.$$

where $h_{p}$ is the piezoelectric layer thickness; $h_{b}$ and $h_{c}$ are the position of the bottom and top boundaries of the piezoelectric layer to the neutral axis, respectively; and $h_{pc}=\left(h_{b}+h_{c}\right)/2$ is the position of the thickness center of the piezoelectric layer to the neutral axis. Defining the geometric ratio $n_{g}=h_{s}/h_{p}$ and elastic ratio $n_{e}=Y_{s}/Y_{p}$, the positions of the layer boundaries of a unimorph harvester to the neutral axis, $h_{a}$, $h_{b}$, $h_{c}$, and $h_{pc}$, could be represented by $n_{g}$, $n_{e}$, and $h_{p}$ as:

(14) $$\left\{ \begin{array}{l} h_{a}=-\frac{h_{p}}{2}\left(\frac{{n_{g}}^{2}n_{e}+2m+1}{n_{g}n_{e}+1}\right)\\ h_{b}=h_{a}+h_{s}=\frac{h_{p}}{2}\left(\frac{{n_{g}}^{2}n_{e}-1}{n_{g}n_{e}+1}\right)\\ h_{c}=h_{b}+h_{p}=\frac{h_{p}}{2}\left(\frac{{n_{g}}^{2}n_{e}+2n_{g}n_{e}+1}{n_{g}n_{e}+1}\right)\\ h_{pc}=\frac{h_{b}+h_{c}}{2}=\frac{h_{p}}{2}\left(\frac{{n_{g}}^{2}n_{e}+n_{g}n_{e}}{n_{g}n_{e}+1}\right) \end{array}\right.$$

Similarly, for bimorph harvesters with series connection, the electric field and electric potential could be solved as:

(15) $$E_{3}\left(x,z,t\right)=\left\{ \begin{array}{l} -\frac{d_{31}Y_{p}}{\varepsilon_{33}^{S}}\frac{\partial^{2}w_{rel}\left(x,t\right)}{\partial x^{2}}\left(z+\frac{h_{p}+h_{s}}{2}\right)-\frac{v\left(t\right)}{2h_{p}},\;\;\;-h_{p}-\frac{h_{s}}{2}\leq z\leq -\frac{h_{s}}{2}\\ \frac{d_{31}Y_{p}}{\varepsilon_{33}^{S}}\frac{\partial^{2}w_{rel}\left(x,t\right)}{\partial x^{2}}\left(z-\frac{h_{p}+h_{s}}{2}\right)-\frac{v\left(t\right)}{2h_{p}},\;\;\;\;\;\frac{h_{s}}{2}\leq z\leq h_{p}+\frac{h_{s}}{2} \end{array}\right.$$

(16) $$\begin{array}{l} \Phi \left(x,z,t\right)=\\ \left\{ \begin{array}{l} -\frac{d_{31}Y_{p}}{\varepsilon_{33}^{S}}\frac{\partial^{2}w_{rel}\left(x,t\right)}{\partial x^{2}}\left[-\frac{z^{2}}{2}-\frac{\left(h_{p}+h_{s}\right)z}{2}-\frac{\left(2h_{p}+h_{s}\right)h_{s}}{8}\right]+\frac{v\left(t\right)}{2h_{p}}\left(z+h_{p}+\frac{h_{s}}{2}\right),\;\;\;-h_{p}-\frac{h_{s}}{2}\leq z\leq -\frac{h_{s}}{2}\\ \frac{d_{31}Y_{p}}{\varepsilon_{33}^{S}}\frac{\partial^{2}w_{rel}\left(x,t\right)}{\partial x^{2}}\left[-\frac{z^{2}}{2}+\frac{\left(h_{p}+h_{s}\right)z}{2}-\frac{\left(2h_{p}+h_{s}\right)h_{s}}{8}\right]+\frac{v\left(t\right)}{2h_{p}}\left(z+h_{p}-\frac{h_{s}}{2}\right),\;\;\;\;\;\frac{h_{s}}{2}\leq z\leq h_{p}+\frac{h_{s}}{2} \end{array}\right. \end{array}$$

For bimorph harvesters with parallel connection, the electric field and electric potential could be solved as:

(17) $$E_{3}\left(x,z,t\right)=\left\{ \begin{array}{l} \frac{d_{31}Y_{p}}{\varepsilon_{33}^{S}}\frac{\partial^{2}w_{rel}\left(x,t\right)}{\partial x^{2}}\left(z+\frac{h_{p}+h_{s}}{2}\right)-\frac{v\left(t\right)}{h_{p}},\;\;\;-h_{p}-\frac{h_{s}}{2}\leq z\leq -\frac{h_{s}}{2}\\ \frac{d_{31}Y_{p}}{\varepsilon_{33}^{S}}\frac{\partial^{2}w_{rel}\left(x,t\right)}{\partial x^{2}}\left(z-\frac{h_{p}+h_{s}}{2}\right)+\frac{v\left(t\right)}{h_{p}},\;\;\;\;\;\frac{h_{s}}{2}\leq z\leq h_{p}+\frac{h_{s}}{2} \end{array}\right.$$

(18) $$\begin{array}{l} \Phi \left(x,z,t\right)=\\ \left\{ \begin{array}{l} \frac{d_{31}Y_{p}}{\varepsilon_{33}^{S}}\frac{\partial^{2}w_{rel}\left(x,t\right)}{\partial x^{2}}\left[-\frac{z^{2}}{2}-\frac{\left(h_{p}+h_{s}\right)z}{2}-\frac{\left(2h_{p}+h_{s}\right)h_{s}}{8}\right]+\frac{v\left(t\right)}{h_{p}}\left(z+h_{p}+\frac{h_{s}}{2}\right),\;\;\;-h_{p}-\frac{h_{s}}{2}\leq z\leq -\frac{h_{s}}{2}\\ \frac{d_{31}Y_{p}}{\varepsilon_{33}^{S}}\frac{\partial^{2}w_{rel}\left(x,t\right)}{\partial x^{2}}\left[-\frac{z^{2}}{2}+\frac{\left(h_{p}+h_{s}\right)z}{2}-\frac{\left(2h_{p}+h_{s}\right)h_{s}}{8}\right]+\frac{v\left(t\right)}{h_{p}}\left(-z+h_{p}+\frac{h_{s}}{2}\right),\;\;\;\;\;\frac{h_{s}}{2}\leq z\leq h_{p}+\frac{h_{s}}{2} \end{array}\right. \end{array}$$

### 2.3. Unified Electromechanical Governing Equations Containing Both Uniform and Non-Uniform Electric Field Assumption

Using the above basic equations combined with different assumptions of the electric field, i.e., uniform electric field and non-uniform electric field, the expression of the internal bending moment for both unimorph and bimorph configurations could be unified as [33,34,39]:

(19) $$M\left(x,t\right)=YI\frac{\partial^{2}w_{rel}\left(x,t\right)}{\partial x^{2}}+\vartheta v\left(t\right)$$

This expression is formulated by calculating the internal bending moment of the beam using the integration of the normal stress on the cross-section, in which YI is the bending stiffness term for short-circuit condition and ϑ is the electromechanical coupling term. For a unimorph harvester, considering a uniform electric field, the bending stiffness term could be expressed using the width of the harvester *b*, the geometric and elastic parameters of the piezoelectric layer $h_{p}$ and $Y_{p}$, the geometric ratio $n_{g}=h_{s}/h_{p},$ and the elastic ratio $n_{e}=Y_{s}/Y_{p}$:

(20) $$YI=YI_{U}=\frac{Y_{p}b{h_{p}}^{3}}{12}\left(\frac{{n_{g}}^{4}{n_{e}}^{2}+4{n_{g}}^{3}n_{e}+6{n_{g}}^{2}n_{e}+4n_{g}n_{e}+1}{n_{g}n_{e}+1}\right)$$

When employing the previously proposed non-uniform electric field, a refined bending stiffness can be derived as:

(21) $$YI=Y{I_{U}}^{\prime }=\frac{Y_{p}b{h_{p}}^{3}}{12}\left(\frac{{n_{g}}^{4}{n_{e}}^{2}+4{n_{g}}^{3}n_{e}+6{n_{g}}^{2}n_{e}+4n_{g}n_{e}+1}{n_{g}n_{e}+1}+\frac{{d_{31}}^{2}Y_{p}}{\varepsilon_{33}^{S}}\right)$$

As for the coupling term of a unimorph harvester, that obtained based on both uniform and non-uniform electric fields could be expressed as:

(22) $$\vartheta =\vartheta_{U}=-\frac{Y_{p}d_{31}bh_{p}}{2}\left(\frac{{n_{g}}^{2}n_{e}+n_{g}n_{e}}{n_{g}n_{e}+1}\right)$$

For a bimorph harvester considering a uniform electric field, whether it is connected in series or parallel, the bending stiffness could be expressed as:

(23) $$YI=YI_{B}=\frac{Y_{p}b{h_{p}}^{3}}{12}\left({n_{g}}^{3}n_{e}+6{n_{g}}^{2}+12n_{g}+8\right)$$

When incorporating the non-uniform electric field proposed previously, the corrected bending stiffness of a bimorph harvester for both series and parallel connections could be derived as:

(24) $$YI=Y{I_{B}}^{\prime }=\frac{Y_{p}b{h_{p}}^{3}}{12}\left({n_{g}}^{3}n_{e}+6{n_{g}}^{2}+12n_{g}+8+\frac{2{d_{31}}^{2}Y_{p}}{\varepsilon_{33}^{S}}\right)$$

As for the coupling term of a bimorph harvester with series connection, that obtained based on both uniform and non-uniform electric fields could be expressed as:

(25) $$\vartheta =\vartheta_{B}=-\frac{Y_{p}d_{31}bh_{p}}{2}\left(n_{g}+1\right)$$

And the coupling term of a parallel connected bimorph harvester is $\vartheta =-2\vartheta_{B}$. It is noteworthy that if the grounded electrodes of a parallel connected bimorph harvester switch to the inner side, this coupling term would become $2\vartheta_{B}$. For different electric field assumptions and different configurations, the expressions of YI and ϑ are summarized in Table 1. If the electrode and piezoelectric layer cover the entire length of the harvester, Equation (19) could be rewritten as

(26) $$M\left(x,t\right)=YI\frac{\partial^{2}w_{rel}\left(x,t\right)}{\partial x^{2}}+\vartheta v\left(t\right)\left[H\left(x\right)-H\left(x-L\right)\right]$$

where *L* is the length of the harvester, and H(x) is the Heaviside function. Substituting Equation (26) into Equation (8) yields the unified mechanical governing equation of motion with electric coupling:

(27) $$\begin{array}{l} YI\frac{\partial^{4}w_{rel}\left(x,t\right)}{\partial x^{4}}+c_{s}I\frac{\partial^{5}w_{rel}\left(x,t\right)}{\partial x^{4}\partial t}+c_{a}\frac{\partial w_{rel}\left(x,t\right)}{\partial t}+m\frac{\partial^{2}w_{rel}\left(x,t\right)}{\partial t^{2}}\\ +\vartheta v\left(t\right)\left[\frac{d\delta \left(x\right)}{dx}-\frac{d\delta \left(x-L\right)}{dx}\right]=-m\frac{\partial^{2}w_{b}\left(x,t\right)}{\partial t^{2}} \end{array}$$

where δ(x) is the Dirac delta function, and it satisfies:

(28) $$\left\{ \begin{array}{l} \delta \left(x\right)=\frac{dH\left(x\right)}{dx}\\ \int_{-\infty }^{\infty }\frac{d^{\left(n\right)}\delta \left(x-x_{0}\right)}{dx^{\left(n\right)}}f\left(x\right)dx={\left(-1\right)}^{n}\frac{df^{\left(n\right)}\left(x_{0}\right)}{dx^{\left(n\right)}} \end{array}\right.$$

For various electric field assumptions and different harvester configurations, the electrical governing equation could be unified by substituting the ungrounded side surface electric displacement, together with its outward unit normal vector, into Equation (9):

(29) $$C_{p}\frac{dv\left(t\right)}{dt}+\frac{v\left(t\right)}{R_{l}}=\vartheta \int_{x=0}^{L}\frac{\partial^{3}w_{rel}\left(x,t\right)}{\partial x^{2}\partial t}dx$$

where $C_{p}$ is the capacitance. For different configurations, $C_{p}$ is presented in Table 1. Based on the expressions for YI, ϑ, and $C_{p}$ shown in Table 1, it should be noticed that when the non-uniform electric field assumption is applied, only the bending stiffness term *YI* is affected, while the other parameters remain unchanged.

### 2.4. Dynamic Response to Harmonic Base Motion

To solve the electromechanically coupled equations given in Equations (27) and (29), the relative transverse deflection $w_{rel}(x,t)$ is represented by an absolutely and uniformly convergent series of the eigenfunctions, expressed as:

(30) $$w_{rel}\left(x,t\right)=\sum_{r=1}^{\infty }\phi_{r}\left(x\right)\eta_{r}\left(t\right)$$

where $\phi_{r}(x)$ is the mass normalized eigenfunction, and $\eta_{r}(t)$ is the modal coordinate of the *r*th mode. For a clamped-free cantilevered beam with no tip mass, $\phi_{r}(x)$ is provided as:

(31) $$\phi_{r}\left(x\right)=\sqrt{\frac{1}{mL}}\left[\cosh\frac{\lambda_{r}}{L}x-\cos\frac{\lambda_{r}}{L}x-\sigma_{r}\left(\sinh\frac{\lambda_{r}}{L}x-\sin\frac{\lambda_{r}}{L}x\right)\right]$$

where $\lambda_{r}$ is the dimensionless frequency numbers obtained from the characteristic equation given by:

(32) 1+cosλcoshλ=0

and $\sigma_{r}$ is expressed as:

(33) $$\sigma_{r}=\frac{\sinh\lambda_{r}-\sin\lambda_{r}}{\cosh\lambda_{r}+\cos\lambda_{r}}$$

Equation (32) can be numerically solved, and the first three orders of $\lambda_{r}$ are obtained as $\lambda_{1}=1.8751$, $\lambda_{2}=4.6941$, and $\lambda_{3}=7.8548$.

The mass normalized eigenfunction $\phi_{r}(x)$ obeys the following orthogonality relations:

(34) $$\left\{ \begin{array}{l} \int_{x=0}^{L}m\phi_{s}\left(x\right)\phi_{r}\left(x\right)dx=\delta_{rs}\\ \int_{x=0}^{L}YI\phi_{s}\left(x\right)\frac{d^{4}\phi_{r}\left(x\right)}{dx^{4}}dx={\omega_{r}}^{2}\delta_{rs} \end{array}\right.$$

where $\delta_{rs}$ is the Kronecker delta, equal to 1 when s=r and 0 otherwise, and $\omega_{r}$ is the short-circuit undamped natural frequency of the *r*th mode, given by:

(35) $$\omega_{r}={\lambda_{r}}^{2}\sqrt{\frac{YI}{mL^{4}}}$$

Substituting Equation (30) into Equations (27) and (29), respectively, considering the orthogonality conditions mentioned in Equation (34), the electromechanical governing equations can be obtained as:

(36) $$\left\{ \begin{array}{l} \frac{d^{2}\eta_{r}\left(t\right)}{dt^{2}}+2\zeta_{r}\omega_{r}\frac{d\eta_{r}\left(t\right)}{dt}+{\omega_{r}}^{2}\eta_{r}\left(t\right)+\chi_{r}v\left(t\right)=-m\left(\gamma_{r}^{w}\frac{d^{2}g\left(t\right)}{dt^{2}}+\gamma_{r}^{\theta }\frac{d^{2}h\left(t\right)}{dt^{2}}\right)\\ \frac{dv\left(t\right)}{dt}+\frac{v\left(t\right)}{R_{l}C_{p}}=\sum_{r=1}^{\infty }\frac{\chi_{r}}{C_{p}}\frac{d\eta_{r}\left(t\right)}{dt} \end{array}\right.$$

where:

(37) $$\left\{ \begin{array}{l} \gamma_{r}^{w}=\int_{x=0}^{L}\phi_{r}\left(x\right)dx\\ \gamma_{r}^{\theta }=\int_{x=0}^{L}x\phi_{r}\left(x\right)dx \end{array}\right.$$

$\chi_{r}$ is the modal coupling term:

(38) $$\chi_{r}={\vartheta \frac{d\phi_{r}\left(x\right)}{dx}|}_{x=L}$$

and $\zeta_{r}$ is the modal damping ratio, expressed as:

(39) $$\zeta_{r}=\frac{c_{s}I\omega_{r}}{2YI}+\frac{c_{a}}{2m\omega_{r}}$$

The modal damping ratio $\zeta_{r}$ is typically determined experimentally in practice, thereby eliminating the need to obtain the values of $c_{s}I$ and $c_{a}$. A detailed discussion on resolving the damping coefficients from the modal damping ratio can be found in reference [33].

If the base motion $w_{b}(x,t)$ is harmonic in the form of $w_{b}\left(x,t\right)=\left(Z_{0}+x\theta_{0}\right)e^{j\omega t}$ (where *j* is the imaginary unit, ω denotes the driving circular frequency, and $Z_{0}$ and $\theta_{0}$ are the amplitude of base translation and rotation, respectively), then the output voltage is also harmonic in the form of $v\left(t\right)=V_{0}e^{j\omega t}$ (where $V_{0}$ is the amplitude of the output voltage). In this case, the electromechanical governing equations given by Equation (36) becomes:

(40) $$\left\{ \begin{array}{l} \frac{d^{2}\eta_{r}\left(t\right)}{dt^{2}}+2\zeta_{r}\omega_{r}\frac{d\eta_{r}\left(t\right)}{dt}+{\omega_{r}}^{2}\eta_{r}\left(t\right)+\chi_{r}V_{0}e^{j\omega t}=m\omega^{2}\left(\gamma_{r}^{w}Z_{0}+\gamma_{r}^{\theta }\theta_{0}\right)e^{j\omega t}\\ \left(j\omega +\frac{1}{R_{l}C_{p}}\right)V_{0}e^{j\omega t}=\sum_{r=1}^{\infty }\frac{\chi_{r}}{C_{p}}\frac{d\eta_{r}\left(t\right)}{dt} \end{array}\right.$$

Solving Equation (40), the steady-state solution of the modal coordinate and the output voltage amplitude can be given as:

(41) $$\eta_{r}\left(t\right)=\frac{\left[m\omega^{2}\left(\gamma_{r}^{w}Z_{0}+\gamma_{r}^{\theta }\theta_{0}\right)-\chi_{r}V_{0}\right]e^{j\omega t}}{{\omega_{r}}^{2}-\omega^{2}+j2\zeta_{r}\omega_{r}\omega }$$

(42) $$V_{0}=\frac{\sum_{r=1}^{\infty }\frac{jm\omega^{3}\chi_{r}R_{l}\left(\gamma_{r}^{w}Z_{0}+\gamma_{r}^{\theta }\theta_{0}\right)}{{\omega_{r}}^{2}-\omega^{2}+j2\zeta_{r}\omega_{r}\omega }}{\sum_{r=1}^{\infty }\frac{j\omega {\chi_{r}}^{2}R_{l}}{{\omega_{r}}^{2}-\omega^{2}+j2\zeta_{r}\omega_{r}\omega }+j\omega R_{l}C_{p}+1}$$

And the relative transverse deflection $w_{rel}(x,t)$ can be easily obtained using Equation (30) and Equation (41). Although $Z_{0}$ and $\theta_{0}$ are real-valued, $v\left(t\right)$ and $w_{b}(x,t)$ do not have to be in phase, which makes $V_{0}$ a complex-valued quantity. Detailed development and resolutions of the electromechanical governing equations can be found in reference [39].

### 2.5. Reduced Single-Mode Response to Harmonic Base Translation

In most cases, the mode of interest is the fundamental vibration mode of the harvester (i.e., r=1), which means that the excitation of the harvester is considered to be around $\omega_{1}$. Accordingly, by simplifying the base motion to a harmonic translation in the transverse direction (i.e., $w_{b}\left(x,t\right)={Z_{0}e}^{j\omega t}$), the reduced expression for the amplitude of the voltage $V_{0}^{*}$ across the external resistance can be written as:

(43) $$V_{0}^{*}=\frac{jm\omega^{3}\chi_{1}\gamma_{1}^{w}R_{l}Z_{0}}{j\omega {\chi_{1}}^{2}R_{l}+\left({\omega_{1}}^{2}-\omega^{2}+j2\zeta_{1}\omega_{1}\omega \right)\left(1+j\omega R_{l}C_{p}\right)}$$

And the absolute value of $V_{0}^{*}$ can be expressed as:

(44) $$\left|V_{0}^{*}\right|=\frac{m\omega^{3}\chi_{1}\gamma_{1}^{w}R_{l}Z_{0}}{\sqrt{{\left[{\omega_{1}}^{2}-\omega^{2}\left(1+2\zeta_{1}\omega_{1}R_{l}C_{p}\right)\right]}^{2}+{\left[2\zeta_{1}\omega_{1}\omega +\omega R_{l}\left({\chi_{1}}^{2}+{\omega_{1}}^{2}C_{p}-\omega^{2}C_{p}\right)\right]}^{2}}}$$

Furthermore, the absolute value of power output can be expressed as:

(45) $$\left|P_{0}^{*}\right|=\frac{{\left|V_{0}^{*}\right|}^{2}}{R_{l}}=\frac{{\left(m\omega^{3}\chi_{1}\gamma_{1}^{w}Z_{0}\right)}^{2}R_{l}}{{\left[{\omega_{1}}^{2}-\omega^{2}\left(1+2\zeta_{1}\omega_{1}R_{l}C_{p}\right)\right]}^{2}+{\left[2\zeta_{1}\omega_{1}\omega +\omega R_{l}\left({\chi_{1}}^{2}+{\omega_{1}}^{2}C_{p}-\omega^{2}C_{p}\right)\right]}^{2}}$$

The formula of the output voltage and output power can be rewritten with respect to dimensionless variables as follows:

(46) $$\left|V_{0}^{*}\right|=\frac{m\gamma_{1}^{w}\omega^{2}Z_{0}\sqrt{R_{l}}}{\sqrt{\omega_{1}}}\frac{\Omega k\sqrt{\alpha }}{\sqrt{{\left[1-\Omega^{2}-2\zeta_{1}\Omega^{2}\alpha \right]}^{2}+{\left[2\zeta_{1}\Omega +\Omega \alpha \left(1-\Omega^{2}+k^{2}\right)\right]}^{2}}}$$

(47) $$\left|P_{0}^{*}\right|=\frac{{\left(m\gamma_{1}^{w}\omega^{2}Z_{0}\right)}^{2}}{\omega_{1}}\frac{\Omega^{2}k^{2}\alpha }{{\left[1-\Omega^{2}-2\zeta_{1}\Omega^{2}\alpha \right]}^{2}+{\left[2\zeta_{1}\Omega +\Omega \alpha \left(1-\Omega^{2}+k^{2}\right)\right]}^{2}}$$

where $\omega^{2}Z_{0}$ represents the amplitude of the base excitation acceleration, and $\zeta_{1}$, Ω, α, and $k^{2}$ are dimensionless variables. Specifically, $\zeta_{1}$ is the damping ratio for the fundamental mode of the harvester, as presented in Equation (39). Ω is the frequency ratio, α is the resistance turning ratio, and $k^{2}$ is the squared effective electromechanical coupling coefficient. Considering the mass normalized eigenfunction adopted in this study, Ω, α, and $k^{2}$ could be represented by the original system parameters as follows:

(48) $$\left\{ \begin{array}{l} \Omega =\frac{\omega }{\omega_{1}}\\ \alpha =R_{l}C_{p}\omega_{1}\\ k^{2}=\frac{{\chi_{1}}^{2}}{C_{p}{\omega_{1}}^{2}} \end{array}\right.$$

Here, the effective electromechanical coupling coefficient squared $k^{2}$ is an important parameter directly reflecting the mechanical-to-electrical energy conversion efficiency of the system. $k^{2}$ describes the effectiveness of quasi-static energy conversion between electrical and mechanical forms. For a cantilevered energy harvester in an open circuit subjected to quasi-static stress, it is equal to the electrostatic energy divided by the total energy in the system. Theoretically, it is also related to the open-circuit undamped natural frequency ($\omega_{1}^{OC}$) and the short-circuit undamped natural frequency ($\omega_{1}^{SC}$ or $\omega_{1}$) as:

(49) $$k^{2}=\frac{{\left(\omega_{1}^{OC}\right)}^{2}-{\left(\omega_{1}^{SC}\right)}^{2}}{{\left(\omega_{1}^{SC}\right)}^{2}}$$

Equation (49) provides a method to experimentally determine the squared effective electromechanical coupling coefficient $k^{2}$ while the damping ratio is far less than 1.0, approximately $\varsigma_{1}<0.2$. 

The optimal resistance turning ratio can be determined by differentiating the power expression Equation (47) with respect to the resistance tuning ratio, letting the derivative be zero, and solving the optimal resistance turning ratio:

(50) $$\alpha_{opt}=\frac{1}{\Omega }\sqrt{\frac{{\left(1-\Omega^{2}\right)}^{2}+{\left(2\zeta_{1}\Omega \right)}^{2}}{{\left(1+k^{2}-\Omega^{2}\right)}^{2}+{\left(2\zeta_{1}\Omega \right)}^{2}}}$$

Substituting Equation (50) into Equation (47), the optimal power as a function of the frequency ratio can be obtained. The curve of the optimal power with respect to the frequency ratio or excitation frequency is considered as a power envelope.

## 3. Verification of the Non-Uniform Electric Field Assumption

In Section 2, considering both uniform and non-uniform electric field assumptions, the electromechanically coupled model of cantilevered piezoelectric energy harvesters is provided. While incorporating the non-uniform electric field, the bending stiffness term YI for both unimorph and bimorph harvesters is explicitly modified as presented in Table 1. To demonstrate that the model incorporating a non-uniform electric field in the piezoelectric layer is more accurate than the traditional model considering a uniform electric field, sample harvesters are employed and a numerical analysis is conducted using both the models, then compared with the results of a finite element analysis.

Herein, a unimorph sample harvester ($H_{p}=0.5\;mm,\;H_{s}=0.5\;mm,\;b=20\;mm,\;L=100\;mm$) and a parallel-connected bimorph sample harvester ($H_{p}=0.5\;mm,\;H_{s}=0,\;b=20\;mm,\;L=100\;mm$) are introduced to demonstrate the performance corrections when incorporating a non-uniform electric field assumption. The sample harvesters are made from PZT-5H and aluminum. In Table 2, the one-dimensional material parameters of aluminum and PZT-5H under plane stress conditions are provided. Detailed resolutions of the one-dimensional material parameters of PZT-5H from three-dimensional material parameters for the plane problem are presented in the Appendix A. As for the damping ratio, we suppose $\zeta_{1}=0.01$. In this section, a reduced single-mode response to harmonic base motion presented in Section 2.5 is employed, and the power output FRF (frequency response function) is defined as the output power $\left|P_{0}^{*}\right|$ divided by squared excitation acceleration ${\left(-\omega^{2}Z_{0}\right)}^{2}$. Power output prediction of these two sample harvesters based on a reduced single-mode model is conducted using MATLAB programs, which are provided in the Supplementary Materials.

The finite element results for reference were achieved using COMCOL Multiphysics 5.6. In the COMSOL project, default PZT-5H material parameters, manually entered aluminum material parameters, a solid mechanics module, a 2D model, and plane stress analysis are employed. To adopt the Rayleigh damping mechanism, the first two orders of the damping ratio and the short-circuit undamped natural frequency are required. Therefore, we suppose $\zeta_{1}=0.01$, $\zeta_{2}=0.02$. And the first two orders of short-circuit undamped natural frequency vary with respect to specific sample harvesters, which can be obtained using steady-state analysis in the COMSOL project by setting the damping ratio and the circuit load resistance to zero. For the unimorph sample harvester in the COMSOL project, $f_{1}=58.307\;Hz$ and $f_{2}=365.24\;Hz$. As for the bimorph sample harvester in the COMSOL project, $f_{1}=46.952\;Hz$ and $f_{2}=294.11\;Hz$.

**Figure 2:**
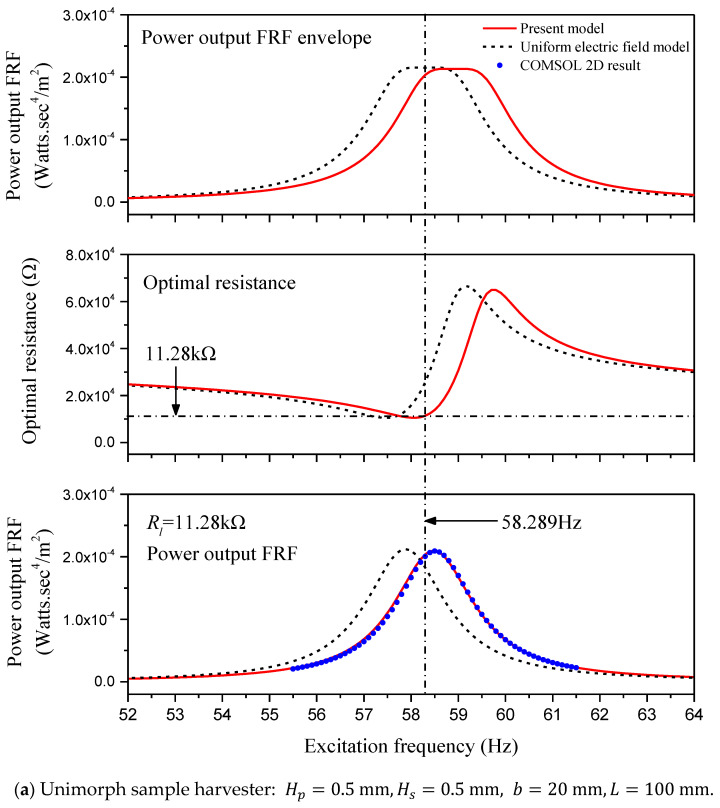

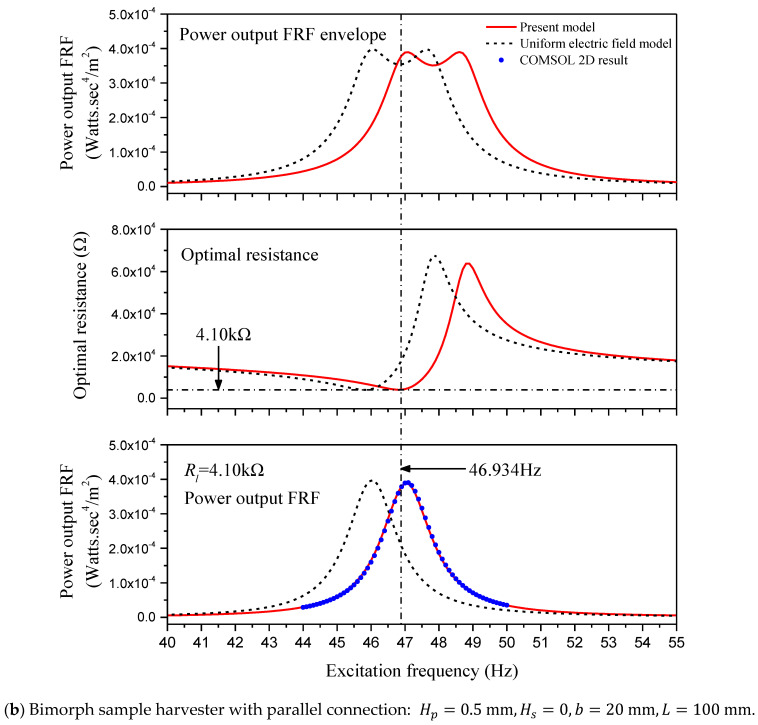
Comparison of power output FRF for sample harvesters.

Numerical results of these two sample harvesters are presented in Figure 2. The first rows of Figure 2a,b represent the envelope of the power output FRF of these two sample harvesters with respect to the excitation frequency obtained based on Equations (47) and (50). The second row is the optimal resistance obtained based on Equation (50). And the last row is the power output FRF, while the load resistance is exactly the optimal resistance for the short-circuit undamped natural frequency $\omega_{1}$. Both the traditional model considering a uniform electric field as well as the improved model incorporating a non-uniform electric field are taken into account. The simulated power output results in COMSOL projects are provided in the last row for verification. 

For the unimorph sample harvester, using the improved model incorporating a non-uniform electric field, the short-circuit undamped natural frequency is $f_{1}=\omega_{1}/\left(2\pi \right)=58.289\;Hz$, which is very close to the COMSOL result, and the corresponding optimal resistance is 11.28 kΩ. As for the parallel connected bimorph sample harvester, the short-circuit undamped natural frequency is $f_{1}=46.934\;Hz$, which is also very close to the COMSOL result, and the corresponding optimal resistance is 4.10 kΩ. For the unimorph sample harvester connected to a 11.28 kΩ load resistance, the power output FRF is presented in the last row of Figure 2a, and that of the bimorph sample harvester connected to a 4.10 kΩ load resistance is presented in the last row of Figure 2b. Whether for a unimorph harvester or a bimorph harvester, it is obvious that the result obtained using the improved model incorporating a non-uniform electric field is basically consistent with the COMSOL 2D result, while a resonant frequency shift comparative to the traditional model is observed.

## 4. Determinants of Performance Corrections

According to the modeling of cantilevered piezoelectric energy harvesters incorporating non-uniform electric fields, the performance corrections to traditional model considering uniform electric fields are ultimately due to the bending stiffness correction, as presented in Table 1. As a key parameter, the bending stiffness term has a great influence on both the static and dynamic performance of cantilevered piezoelectric composites. For unimorph harvesters, the bending stiffness term ${YI}_{U}$ (Equation (20)) is modified to ${{YI}_{U}}^{\prime }$ (Equation (21)). Similarly, for series- and parallel-connected bimorph harvesters, the bending stiffness term ${YI}_{B}$ (Equation (23)) is modified to ${{YI}_{B}}^{\prime }$ (Equation (24)). The respective correction factors for the bending stiffness of unimorph and bimorph harvesters can be defined as:

(51) $$\begin{array}{l} \delta_{U}=\frac{Y{I_{U}}^{\prime }}{YI_{U}}=1+\frac{{d_{31}}^{2}Y_{p}}{\varepsilon_{33}^{S}}\left(\frac{n_{g}n_{e}+1}{{n_{g}}^{4}{n_{e}}^{2}+4{n_{g}}^{3}n_{e}+6{n_{g}}^{2}n_{e}+4n_{g}n_{e}+1}\right)\\ \delta_{B}=\frac{Y{I_{B}}^{\prime }}{YI_{B}}=1+\frac{{d_{31}}^{2}Y_{p}}{\varepsilon_{33}^{S}}\left(\frac{2}{{n_{g}}^{3}n_{e}+6{n_{g}}^{2}+12n_{g}+8}\right) \end{array}$$

Recall that the electromechanical coupling coefficient of piezoelectric materials is defined as:

(52) $$k_{31}=\frac{d_{31}}{\sqrt{\varepsilon_{33}^{T}s_{11}^{E}}}$$

Equation (51) can be rewritten as:

(53) $$\begin{array}{l} \delta_{U}=\frac{Y{I_{U}}^{\prime }}{YI_{U}}=1+\frac{{k_{31}}^{2}}{1-{k_{31}}^{2}}\left(\frac{n_{g}n_{e}+1}{{n_{g}}^{4}{n_{e}}^{2}+4{n_{g}}^{3}n_{e}+6{n_{g}}^{2}n_{e}+4n_{g}n_{e}+1}\right)\\ \delta_{B}=\frac{Y{I_{B}}^{\prime }}{YI_{B}}=1+\frac{{k_{31}}^{2}}{1-{k_{31}}^{2}}\left(\frac{2}{{n_{g}}^{3}n_{e}+6{n_{g}}^{2}+12n_{g}+8}\right) \end{array}$$

**Figure 3:**
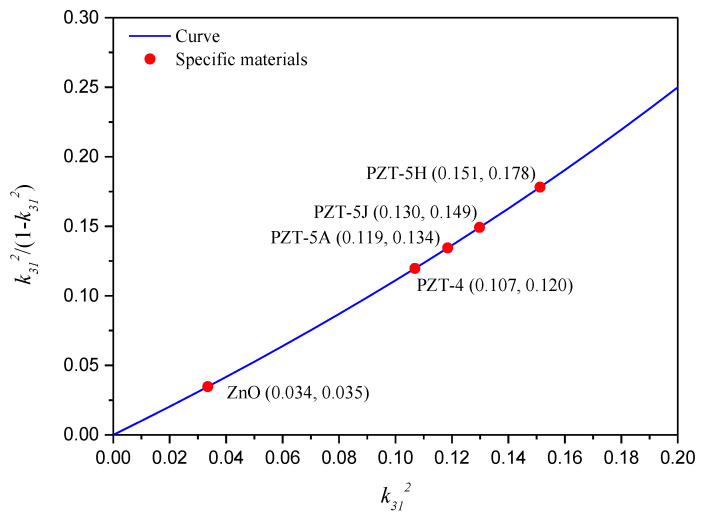
Impact of material electromechanical coupling strength.

Through Equation (53), it is found that the bending stiffness correction and the resulting performance correction are only related to three dimensionless parameters, i.e., the geometric ratio $n_{g}=h_{s}/h_{p}$, the elastic ratio $n_{e}=Y_{s}/Y_{p}$, and the electromechanical coupling coefficient squared ${k_{31}}^{2}$. The impact of ${k_{31}}^{2}$ on the bending stiffness correction factor $\delta_{U}$ and $\delta_{B}$ are reflected by the same term ${k_{31}}^{2}/\left(1-{k_{31}}^{2}\right)$, and the value of this term with respect to ${k_{31}}^{2}$ is presented in Figure 3. Theoretically, ${k_{31}}^{2}$ is less than 1.0, but generally, ${k_{31}}^{2}$ is less than 0.2 for common piezoelectric materials. As we can see in Figure 3, the value of ${k_{31}}^{2}/\left(1-{k_{31}}^{2}\right)$ monotonically increases with the increase in ${k_{31}}^{2}$. This means that, for the given geometric ratio $n_{g}$ and elastic ratio $n_{e}$, the larger ${k_{31}}^{2}$ is, the larger $\delta_{U}$, $\delta_{B}$, and the resulting performance correction become. The values of ${k_{31}}^{2}$ and ${k_{31}}^{2}/\left(1-{k_{31}}^{2}\right)$ for common piezoelectric materials are pointed out in Figure 3.

**Figure 4:**
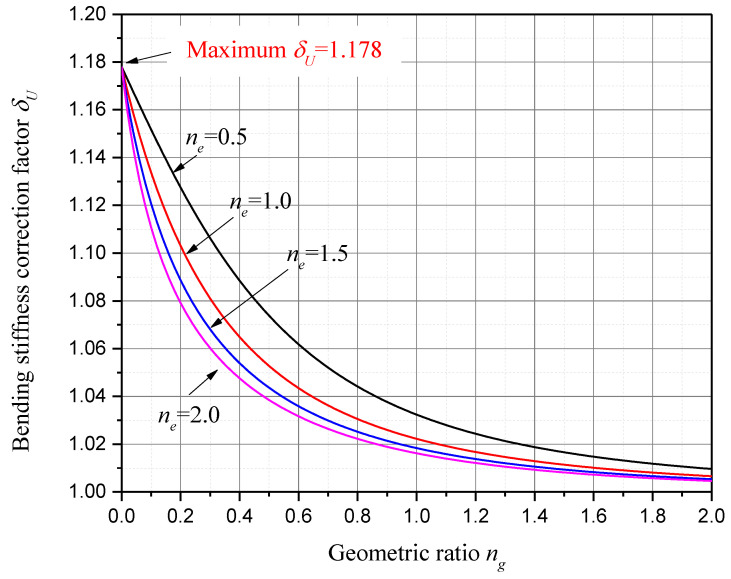
Bending stiffness correction factor $\delta_{U}$ with respect to geometric ratio $n_{g}$ and elastic ratio $n_{e}$ (PZT-5H unimorph harvesters).

**Figure 5:**
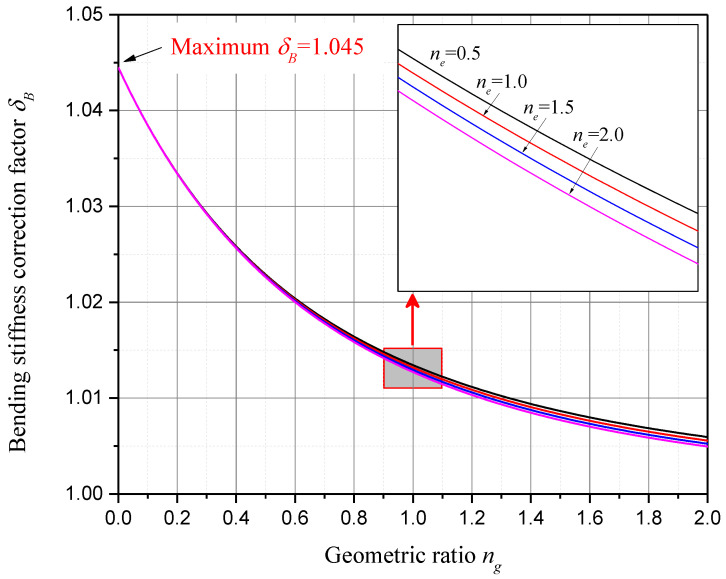
Bending stiffness correction factor $\delta_{B}$ with respect to geometric ratio $n_{g}$ and elastic ratio $n_{e}$ (PZT-5H bimorph harvesters).

For a specific piezoelectric material, for example, PZT-5H, whose electromechanical coupling coefficient squared ${k_{31}}^{2}=0.151$, the bending stiffness correction factor of unimorph harvester $\delta_{U}$ with respect to geometric ratio $n_{g}$ and elastic ratio $n_{e}$ is presented in Figure 4. And the bending stiffness correction factor of bimorph harvester $\delta_{B}$ with respect to $n_{g}$ and $n_{e}$ is presented in Figure 5. In both Figure 4 and Figure 5, four discrete elastic ratios $n_{e}$ and continuous geometric ratios $n_{g}$ are considered, since the elastic parameter of the substrate is usually limited to a few commonly used materials, while the geometric dimensions have many variations. 

As we can see in Figure 4 and Figure 5, it is found that both $\delta_{U}$ and $\delta_{B}$ are constantly greater than 1.0, and they decrease monotonically as $n_{g}$ or $n_{e}$ increases. While $n_{g}$ is larger than 2.0, both $\delta_{U}$ and $\delta_{B}$ are less than 1.01. This means that, when the substructure layer’s thickness is over twice the piezoelectric layer’s thickness, the bending stiffness corrections for the PZT-5H unimorph and bimorph harvesters are less than 1%. This result is obtained when $n_{e}$ is in the range from 0.5 to 2.0, which covers most of the commonly used substrate materials.

On the other hand, for PZT-5H unimorph harvesters, while $n_{g}$ decreases to zero, a maximum bending stiffness correction factor $\delta_{U}=1.178$ is observed, which indicates a 17.8% bending stiffness correction and an 8.5% short-circuit undamped natural frequency correction. For PZT-5H bimorph harvesters, while $n_{g}$ decreases to zero, a maximum bending stiffness correction factor $\delta_{B}=1.045$ is observed, which indicates a 4.5% bending stiffness correction and a 2.2% short-circuit undamped natural frequency correction. From Figure 4 and Figure 5, we know that a maximum bending stiffness correction factor can be achieved while the substructure layer thickness is zero. Then, substituting $n_{g}=0$ into Equation (53), the maximum bending stiffness correction factor is obtained as

(54) $$\begin{array}{l} \delta_{U}^{Max}=1+\frac{{k_{31}}^{2}}{1-{k_{31}}^{2}}\\ \delta_{B}^{Max}=1+\frac{{k_{31}}^{2}}{4\left(1-{k_{31}}^{2}\right)} \end{array}$$

**Figure 6:**
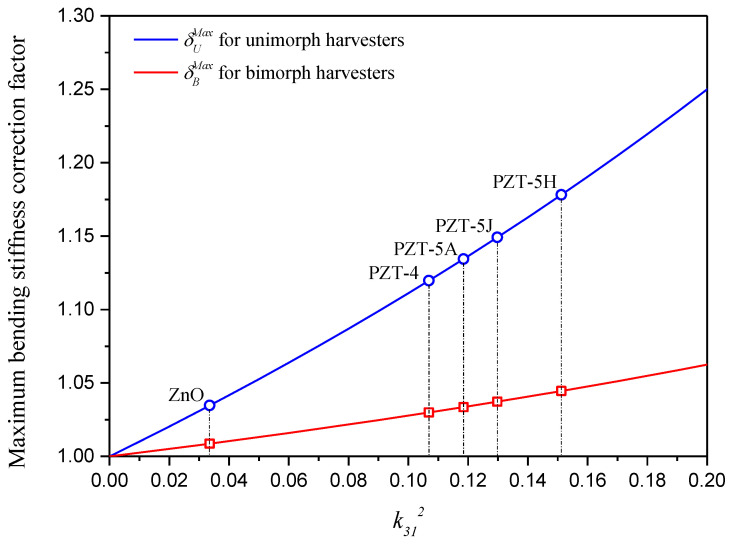
Maximum bending stiffness correction factors $\delta_{U}^{Max}$ and $\delta_{B}^{Max}$ with respect to ${k_{31}}^{2}$.

Through Equation (54), it is found that the achievable maximum bending stiffness correction and resulting maximum performance correction are only related to one dimensionless parameter, i.e., the electromechanical coupling coefficient squared, ${k_{31}}^{2}$. The maximum bending stiffness correction factors $\delta_{U}^{Max}$ and $\delta_{B}^{Max}$ with respect to ${k_{31}}^{2}$ are presented in Figure 6. Although the maximum correction factors for unimorph and bimorph harvesters are both achieved while the harvester consists of only piezoelectric material, $\delta_{U}^{Max}$ is obviously much larger than $\delta_{B}^{Max}$ at the same ${k_{31}}^{2}$. This is due to different internal electric field distributions.

Among commonly used piezoelectric materials, as pointed out in Figure 3 and Figure 6, PZT-5H has the largest electromechanical coupling coefficient squared, i.e., ${k_{31}}^{2}=0.151$, and the corresponding maximum bending stiffness correction factors are as follows: $\delta_{U}^{Max}=1.178$ and $\delta_{B}^{Max}=1.045$. If ${k_{31}}^{2}$ increases to 0.2, which is very likely for PZT-based composites such as macro-fiber composites, the maximum bending stiffness correction factors would become $\delta_{U}^{Max}=1.250$ and $\delta_{B}^{Max}=1.063$. This indicates a 25% maximum bending stiffness correction and a resulting 11.8% maximum short-circuit undamped natural frequency correction for unimorph harvesters, as well as a 6.3% maximum bending stiffness correction and a resulting 3.1% maximum short-circuit undamped natural frequency correction for bimorph harvesters.

## 5. Material Parameters Identification Considering Non-Uniform Electric Field

In Section 3 and Section 4, the improved electromechanical model considering a non-uniform electric field in the piezoelectric layer is utilized to better predict the performance of cantilevered piezoelectric energy harvesters, in which case the material parameters are given in advance. Another scenario in which the improved model can make a difference is the identification of material parameters.

**Figure 7:**
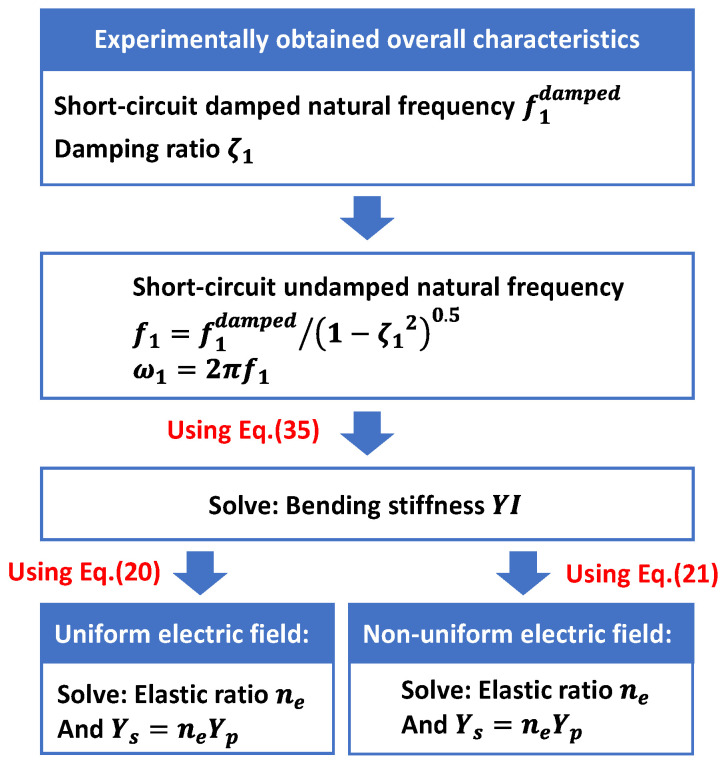
Procedures to identify the Young modulus $Y_{s}$ of the substructure.

Case I: Only the Young modulus $Y_{s}$ of the substructure needs to be identified. In this case, the unimorph sample harvester introduced in Section 3 is employed (a PZT-5H and aluminum unimorph harvester: $H_{p}=0.5mm,\;H_{s}=0.5mm,\;b=20mm,\;L=100mm$). It is assumed that the material parameters of the piezoelectric layer, the geometric dimensions of the harvester, and the mass density of the substructure are known as listed in Table 2, and the Young modulus $Y_{s}$ of the substructure needs to be identified using the experimentally obtained overall characteristics of the energy harvester. The procedures to identify $Y_{s}$ are presented in Figure 7. In this case, the damping ratio $\zeta_{1}$ and the short-circuit damped natural frequency $f_{1}^{damped}$ are experimentally determined in advance.

In order to demonstrate the accuracy of the material parameters identification considering a non-uniform electric field, a numerical experiment is conducted using the two-dimensional COMSOL project, and the theoretically identified $Y_{s}$ is compared with the one originally set in the COMSOL project.

From the numerical experiment, the short-circuit undamped natural frequency of the unimorph sample harvester is obtained as $f_{1}=58.307\;Hz$. For a real unimorph harvester, its damping ratio $\zeta_{1}$ and short-circuit damped natural frequency $f_{1}^{damped}$ can be obtained through a free vibration test, and the corresponding short-circuit undamped natural frequency could be obtained as $f_{1}=f_{1}^{damped}/{\left(1-{\zeta_{1}}^{2}\right)}^{0.5}$. If $\zeta_{1}$ is very small, $f_{1}$ and $f_{1}^{damped}$ would be very close to each other. Combining the mass density and cross-section area of the unimorph harvester, its mass per unit length can be calculated as m=0.102 kg/m. Substituting $\omega_{1}=58.307\times 2\pi rad/s$, L=0.1 m and m=0.102 kg/m into Equation (35), the bending stiffness YI can be solved as $0.1107\;N\cdot m^{2}$.

Based on a traditional uniform electric field, substituting $Y_{p},\;b,\;h_{p},\;n_{g},\;YI$ into Equation (20), the elastic ratio can be solved as $n_{e}=1.2071$, and the Young modulus $Y_{s}$ of the substructure can be identified as $Y_{s}=n_{e}Y_{p}=73.16\;GPa$. Based on a present non-uniform electric field, substituting $Y_{p},\;b,\;h_{p},\;n_{g},d_{31},\;\varepsilon_{33}^{S},\;YI$ into Equation (21), the elastic ratio can be solved as $n_{e}=1.1565$, and the Young modulus $Y_{s}$ of the substructure can be identified as $Y_{s}=n_{e}Y_{p}=70.10\;GPa$. Note that the original set $Y_{s}=70\;GPa$, the identified $Y_{s}$, and the corresponding error are presented in Table 3. According to Table 3, the error in material parameters identification was reduced from 4.51% to 0.14% when the non-uniform electric field was considered.

**Figure 8:**
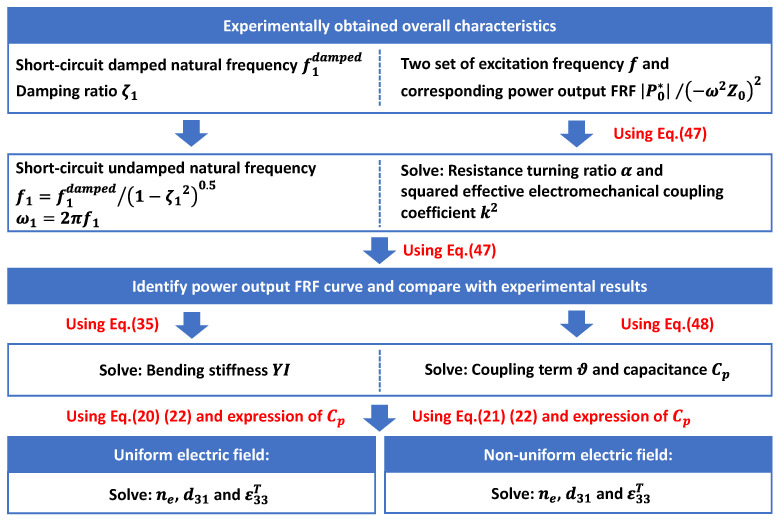
Procedures to identify $Y_{p},\;d_{31},$ and $\varepsilon_{33}^{T}$.

Case II: The material parameters of the piezoelectric layer, $Y_{p},\;d_{31},\;\varepsilon_{33}^{T}$, need to be identified. This case is relatively complicated compared to Case I. In this case, the unimorph sample harvester introduced in the first case is also employed. It is assumed that the geometric dimensions, the material parameters of the substrate layer, and the mass density of the piezoelectric layer are known as listed in Table 2, and the material parameters of the piezoelectric layer $Y_{p},\;d_{31},\;\varepsilon_{33}^{T}$ need to be identified using the experimentally obtained overall characteristics of the energy harvester. The procedures to identify $Y_{p},\;d_{31},\;\varepsilon_{33}^{T}$ are presented in Figure 8. In this case, the damping ratio $\zeta_{1}$, the short-circuit damped natural frequency $f_{1}^{damped}$, and two sets of excitation frequencies f with corresponding power outputs FRF $\left|P_{0}^{*}\right|/{\left(-\omega^{2}Z_{0}\right)}^{2}$ need to be experimentally determined in advance. Specifically, in the present study, these characteristics of the unimorph sample harvester are gathered through a numerical experiment conducted using the two-dimensional COMSOL project. And the following identified material parameters are compared with the ones originally set in the COMSOL project.

**Figure 9:**
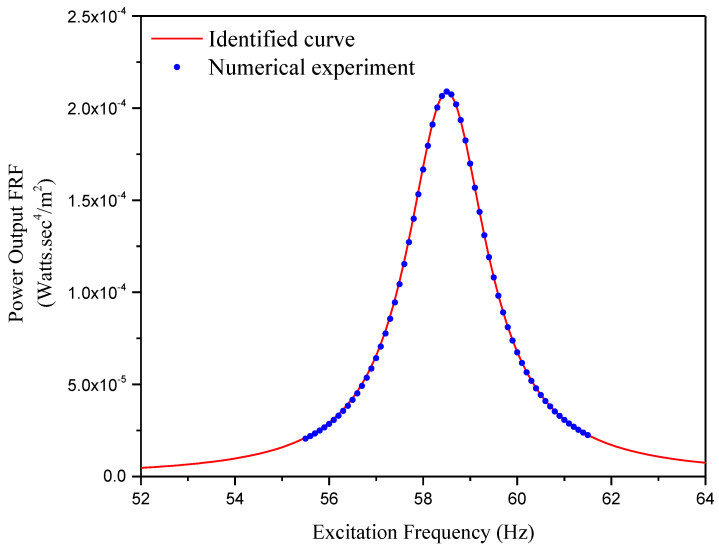
Numerical experiment result and identified curve.

Firstly, we need to identify the power output FRF curve of this unimorph sample harvester using its experimentally obtained overall characteristics. According to Equation (47), four parameters are required, i.e., $f_{1}$, $\zeta_{1}$, $k^{2}$, and α. In the numerical experiment, the short-circuit undamped natural frequency is determined as $f_{1}=58.307\;Hz$, and the damping ratio $\zeta_{1}$ is considered as 0.01. These two parameters can be obtained through free vibration test for a real unimorph harvester, which was described in the first case. The resolution of the other two parameters, $k^{2}$ and α, needs two sets of excitation frequency and corresponding power output FRF to be substituted, respectively, into Equation (47). With the circuit load resistance $R_{l}=11.28\;k\Omega$, the power output FRF of this unimorph sample harvester with respect to the driving frequency is shown in the third row of Figure 2a as a set of blue dots. Take two sets of these blue dots, i.e., $f=\left[58.5,\;59.5\right]\;Hz$, $\left|P_{0}^{*}\right|/{\left(-\omega^{2}Z_{0}\right)}^{2}=\left[2.090,\;1.081\right]\times 10^{-4}\;Ws^{4}/m^{2}$. The substitution of these two sets of data into Equation (47) generates two algebra equations, and the unknown parameters can be solved as $k^{2}=0.0402$ and α=0.4484. Recall the damping ratio $\zeta_{1}=0.01$ and the short-circuit undamped natural frequency $\omega_{1}=58.307\times 2\pi \;rad/s$. The power output FRF of this unimorph sample harvester with respect to the excitation frequency can be identified using Equation (47) and is presented in Figure 9.

Since the power output FRF curve has been successfully identified as shown in Figure 9, the determined value of the resistance turning ratio α and the effective electromechanical coupling coefficient squared $k^{2}$ should be trustworthy, i.e., α=0.4484 and $k^{2}=0.0402$. Furthermore, the circuit load resistance $R_{l}=11.28\;k\Omega$, the short-circuit undamped natural circular frequency $\omega_{1}=58.307\times 2\pi \;rad/s$, the capacitance $C_{p}$, and the coupling term ϑ of this unimorph harvester can be solved using Equation (48), which gives $C_{p}=1.0850\times 10^{-7}\;F$ and $\vartheta =8.8757\times 10^{-5}\;Nm/V$. Recall that the bending stiffness was identified in the first case as $YI=0.1107\;N\cdot m^{2}$. The expressions of YI, ϑ, and $C_{p}$ actually formed three algebra equations containing three unknown constants, i.e., $n_{e}$, $d_{31}$, and $\varepsilon_{33}^{T}$. While the traditional uniform electric field is adopted, these three algebra equations can be summarized as:

(55) $$\left\{ \begin{array}{l} YI=\frac{Y_{s}b{h_{p}}^{3}}{12n_{e}}\left(\frac{{n_{e}}^{2}+14n_{e}+1}{n_{e}+1}\right)\\ \vartheta =-\frac{Y_{s}d_{31}bh_{p}}{n_{e}+1}\\ C_{p}=\frac{\left(n_{e}\varepsilon_{33}^{T}-{d_{31}}^{2}Y_{s}\right)bL}{h_{p}n_{e}} \end{array}\right.$$

The unknown constants can be solved as:

(56) $$\left\{ \begin{array}{l} n_{e}=1.1092\\ d_{31}=-2.6744\times 10^{-10}V/m\\ \varepsilon_{33}^{T}=3575\times 8.85\times 10^{-12}F/m \end{array}\right.$$

And the Young modulus of the piezoelectric layer can be obtained as $Y_{p}=Y_{s}/n_{e}=63.11\;GPa$. While the present non-uniform electric field is incorporated, these three algebra equations can be summarized as:

(57) $$\left\{ \begin{array}{l} YI=\frac{Y_{s}b{h_{p}}^{3}}{12n_{e}}\left(\frac{{n_{e}}^{2}+14n_{e}+1}{n_{e}+1}+\frac{{d_{31}}^{2}Y_{s}}{n_{e}\varepsilon_{33}^{T}-{d_{31}}^{2}Y_{s}}\right)\\ \vartheta =-\frac{Y_{s}d_{31}bh_{p}}{n_{e}+1}\\ C_{p}=\frac{\left(n_{e}\varepsilon_{33}^{T}-{d_{31}}^{2}Y_{s}\right)bL}{h_{p}n_{e}} \end{array}\right.$$

The unknown constants can be solved as:

(58) $$\left\{ \begin{array}{l} n_{e}=1.1515\\ d_{31}=-2.7280\times 10^{-10}V/m\\ \varepsilon_{33}^{T}=3576\times 8.85\times 10^{-12}F/m \end{array}\right.$$

And the Young modulus of the piezoelectric layer can be determined as $Y_{p}=Y_{s}/n_{e}=60.79\;GPa$.

In summary, the material parameters originally set in the numerical experiment and the identified material parameters are presented and compared in Table 4. As we can see in Table 4, while the non-uniform electric field is incorporated, the identification error of the permittivity at constant stress $\varepsilon_{33}^{T}$ is hardly affected. However, the identification error of the Young modulus $Y_{p}$ is reduced from 4.12% to 0.30%, and that of the piezoelectric constant $d_{31}$ is reduced from 2.39% to 0.44%. The above procedures to identify material parameters are realized using MATLAB programs, which are provided in the Supplementary Materials.

## 6. Conclusions

Considering both unimorph and bimorph harvesters, an electromechanically coupled model incorporating a non-uniform electric field in the piezoelectric layer is presented and simplified to a more practical single-mode model. The present model is validated by comparing its power output prediction with that obtained using the COMSOL project. The results show that, when the non-uniform electric field in the piezoelectric layer is incorporated, the accuracy of both the performance prediction and the material parameters identification for cantilevered piezoelectric energy harvesters is improved.

As an improvement to the traditional model, we found that the bending stiffness term is modified explicitly while the non-uniform electric field is employed. Except for the bending stiffness term, other key parameters involved in the electromechanical governing equations, including ϑ and $C_{p}$, stay the same compared to the traditional model. This means that the overall performance corrections are ultimately due to bending stiffness corrections. Interestingly, it was found that the correction of the bending stiffness is related to only three dimensionless parameters, i.e., the geometric ratio $n_{g}=h_{s}/h_{p}$, the elastic ratio $n_{e}=Y_{s}/Y_{p}$, and the piezoelectric materials’ electromechanical coupling coefficient squared ${k_{31}}^{2}$.

For energy harvesters with various piezoelectric materials and geometric dimensions, the upper-limit performance correction is only related to the electromechanical coupling coefficient squared ${k_{31}}^{2}$. If ${k_{31}}^{2}$ increases to 0.2, a 25% maximum bending stiffness correction and a resulting 11.8% maximum short-circuit undamped natural frequency correction for unimorph harvesters, as well as a 6.3% maximum bending stiffness correction and a resulting 3.1% maximum short-circuit undamped natural frequency correction for bimorph harvesters, are observed.

As an inverse problem, the influence of accounting for the non-uniform electric field in the identification of material parameters is also investigated. Procedures to identify material parameters through overall experimentally obtained characteristics are proposed in the present study. Based on a finite element simulation, the material parameters of a unimorph sample harvester are identified and compared with the initially set values. It is found that, with the help of the present model incorporating a non-uniform electric field, the identification errors of $Y_{s}$, $Y_{p}$, and $d_{31}$ were reduced from 4.51%, 4.12%, and 2.39% to 0.14%, 0.30%, and 0.44%, respectively.

## Figures and Tables

**Table 1:** Expressions of YI, ϑ, and $C_{p}$ considering multiple configurations and different electric field assumptions.

		**Unimorph**	Bimorph with Series Connection	Bimorph with Parallel Connection
Based on uniform electric field	YI	${YI}_{U}$ see Equation(20)	${YI}_{B}$ see Equation(23)	${YI}_{B}$
	ϑ	$\vartheta_{U}$ see Equation(22)	$\vartheta_{B}$ see Equation(25)	$-2\vartheta_{B}$
	$C_{p}$	$\frac{\varepsilon_{33}^{S}bL}{h_{p}}$	$\frac{\varepsilon_{33}^{S}bL}{2h_{p}}$	$\frac{{2\varepsilon }_{33}^{S}bL}{h_{p}}$
Based on non-uniform electric field	YI	${{YI}_{U}}^{\prime }$ see Equation(21)	${{YI}_{B}}^{\prime }$ see Equation(24)	${{YI}_{B}}^{\prime }$
	ϑ	$\vartheta_{U}$	$\vartheta_{B}$	$-2\vartheta_{B}$
	$C_{p}$	$\frac{\varepsilon_{33}^{S}bL}{h_{p}}$	$\frac{\varepsilon_{33}^{S}bL}{2h_{p}}$	$\frac{{2\varepsilon }_{33}^{S}bL}{h_{p}}$

**Table 2:** One-dimensional material parameters of PZT-5H and aluminum under plane stress conditions.

PZT-5H elastic compliance at constant electric field, $s_{11}^{E}$ (pm^2^/N)	16.5
PZT-5H mass density, $\rho_{p}$ (kg/m^3^)	7500
PZT-5H piezoelectric constant, $d_{31}$ (pm/V)	−274
PZT-5H permittivity at constant stress, $\varepsilon_{33}^{T}$ (pF/m)	3400 × 8.85
Aluminum Young modulus, $Y_{s}$ (GPa)	70
Aluminum mass density, $\rho_{s}$ (kg/m^3^)	2700

**Table 3:** Identified $Y_{s}$ and corresponding error.

	Identified $Y_{s}$ (GPa)	Error (%)
Based on uniform electric field assumption	73.16 GPa	4.51%
Based on non-uniform electric field assumption	70.10 GPa	0.14%

**Table 4:** Identified $Y_{p}$, $d_{31}$, $\varepsilon_{33}^{T}$, and corresponding error.

		$Y_{p}$ (GPa)	$d_{31}$ (pm/V)	$\varepsilon_{33}^{T}$ (pF/m)
Originally set value in the numerical experiment		60.61	−274	3400 × 8.85
Identified results based on uniform electric field	Value	63.11	−267.44	3575 × 8.85
	Error	4.12%	2.39%	5.15%
Identified results based on non-uniform electric field	Value	60.79	−272.80	3576 × 8.85
	Error	0.30%	0.44%	5.18%

## Data Availability

The datasets used for generating the plots and results in the present study can be directly obtained from the MATLAB and COMSOL projects provided in the Supplementary Materials.

## Supplementary Materials

The following supporting information can be downloaded at: https://www.mdpi.com/article/10.3390/s24154943/s1.

## Appendix A

Suppose axes 1, 2, and 3 represent the *x*, *y*, and *z* axes, respectively. For transversely isotropic piezoelectric materials polarized along the *z*-axis, the d-type three-dimensional constitutive equations can be written as

(A1) $$\left\{\begin{array}{l} S_{1}\\ S_{2}\\ S_{3}\\ 2S_{23}\\ 2S_{31}\\ 2S_{12} \end{array}\right\}=\left[\begin{array}{cccccc} s_{11}^{E} & s_{12}^{E} & s_{13}^{E} & 0 & 0 & 0\\ s_{12}^{E} & s_{11}^{E} & s_{13}^{E} & 0 & 0 & 0\\ s_{13}^{E} & s_{13}^{E} & s_{33}^{E} & 0 & 0 & 0\\ 0 & 0 & 0 & s_{44}^{E} & 0 & 0\\ 0 & 0 & 0 & 0 & s_{44}^{E} & 0\\ 0 & 0 & 0 & 0 & 0 & s_{66}^{E} \end{array}\right]\left\{\begin{array}{l} T_{1}\\ T_{2}\\ T_{3}\\ T_{23}\\ T_{31}\\ T_{12} \end{array}\right\}+\left[\begin{array}{ccc} 0 & 0 & d_{31}\\ 0 & 0 & d_{31}\\ 0 & 0 & d_{33}\\ 0 & d_{15} & 0\\ d_{15} & 0 & 0\\ 0 & 0 & 0 \end{array}\right]\left\{\begin{array}{l} E_{1}\\ E_{2}\\ E_{3} \end{array}\right\}$$

(A2) $$\left\{\begin{array}{l} D_{1}\\ D_{2}\\ D_{3} \end{array}\right\}=\left[\begin{array}{cccccc} 0 & 0 & 0 & 0 & d_{15} & 0\\ 0 & 0 & 0 & d_{15} & 0 & 0\\ d_{31} & d_{31} & d_{33} & 0 & 0 & 0 \end{array}\right]\left\{\begin{array}{l} T_{1}\\ T_{2}\\ T_{3}\\ T_{23}\\ T_{31}\\ T_{12} \end{array}\right\}+\left[\begin{array}{ccc} \varepsilon_{11}^{T} & 0 & 0\\ 0 & \varepsilon_{11}^{T} & 0\\ 0 & 0 & \varepsilon_{33}^{T} \end{array}\right]\left\{\begin{array}{l} E_{1}\\ E_{2}\\ E_{3} \end{array}\right\}$$

The above constitutive equations can be simplified to two-dimensional ones for the plane problem. Specifically for the plane strain problem, substituting $S_{2}=S_{23}=S_{12}=0$ and $E_{2}=0$ into Equations (A1) and (A2) yields:

(A3) $$\begin{array}{l} \left\{\begin{array}{l} S_{1}\\ S_{3}\\ 2S_{31} \end{array}\right\}=\left[\begin{array}{ccc} \bar{s_{11}^{E}} & \bar{s_{13}^{E}} & 0\\ \bar{s_{13}^{E}} & \bar{s_{33}^{E}} & 0\\ 0 & 0 & \bar{s_{44}^{E}} \end{array}\right]\left\{\begin{array}{l} T_{1}\\ T_{3}\\ T_{31} \end{array}\right\}+\left[\begin{array}{cc} 0 & \bar{d_{31}}\\ 0 & \bar{d_{33}}\\ \bar{d_{15}} & 0 \end{array}\right]\left\{\begin{array}{l} E_{1}\\ E_{3} \end{array}\right\}\\ \left\{\begin{array}{l} D_{1}\\ D_{3} \end{array}\right\}=\left[\begin{array}{ccc} 0 & 0 & \bar{d_{15}}\\ \bar{d_{31}} & \bar{d_{33}} & 0 \end{array}\right]\left\{\begin{array}{l} T_{1}\\ T_{3}\\ T_{31} \end{array}\right\}+\left[\begin{array}{cc} \bar{\varepsilon_{11}^{T}} & 0\\ 0 & \bar{\varepsilon_{33}^{T}} \end{array}\right]\left\{\begin{array}{l} E_{1}\\ E_{3} \end{array}\right\} \end{array}$$

where $\bar{s_{11}^{E}}=s_{11}^{E}-\frac{{s_{12}^{E}}^{2}}{s_{11}^{E}}$, $\bar{s_{13}^{E}}=s_{13}^{E}-\frac{s_{12}^{E}s_{13}^{E}}{s_{11}^{E}}$, $\bar{s_{33}^{E}}=s_{33}^{E}-\frac{{s_{13}^{E}}^{2}}{s_{11}^{E}}$, $\bar{s_{44}^{E}}=s_{44}^{E}$, $\bar{d_{31}}=d_{31}-\frac{{d_{31}s}_{12}^{E}}{s_{11}^{E}}$, $\bar{d_{33}}=d_{33}-\frac{d_{31}s_{13}^{E}}{s_{11}^{E}}$, $\bar{d_{15}}=d_{15}$, $\bar{\varepsilon_{11}^{T}}=\varepsilon_{11}^{T}$, and $\bar{\varepsilon_{33}^{T}}=\varepsilon_{33}^{T}-\frac{{d_{31}}^{2}}{s_{11}^{E}}$. For the plane stress problem, substituting $T_{2}=T_{23}=T_{12}=0$ and $E_{2}=0$ into Equations (A1) and (A2) yields:

(A4) $$\begin{array}{l} \left\{\begin{array}{l} S_{1}\\ S_{3}\\ 2S_{31} \end{array}\right\}=\left[\begin{array}{ccc} s_{11}^{E} & s_{13}^{E} & 0\\ s_{13}^{E} & s_{33}^{E} & 0\\ 0 & 0 & s_{44}^{E} \end{array}\right]\left\{\begin{array}{l} T_{1}\\ T_{3}\\ T_{31} \end{array}\right\}+\left[\begin{array}{cc} 0 & d_{31}\\ 0 & d_{33}\\ d_{15} & 0 \end{array}\right]\left\{\begin{array}{l} E_{1}\\ E_{3} \end{array}\right\}\\ \left\{\begin{array}{l} D_{1}\\ D_{3} \end{array}\right\}=\left[\begin{array}{ccc} 0 & 0 & d_{15}\\ d_{31} & d_{33} & 0 \end{array}\right]\left\{\begin{array}{l} T_{1}\\ T_{3}\\ T_{31} \end{array}\right\}+\left[\begin{array}{cc} \varepsilon_{11}^{T} & 0\\ 0 & \varepsilon_{33}^{T} \end{array}\right]\left\{\begin{array}{l} E_{1}\\ E_{3} \end{array}\right\} \end{array}$$

For the Euler–Bernoulli beam model adopted in the present study, an additional assumption $T_{3}=0$ can be applied and substituted into Equations (A3) and (A4), which lead to the one-dimensional constitutive equations of piezoelectric materials for the plane strain and plane stress problems, respectively:

(A5) $$\left\{ \begin{array}{l} S_{1}=\bar{s_{11}^{E}}T_{1}+\bar{d_{31}}E_{3}\\ D_{3}=\bar{d_{31}}T_{1}+\bar{\varepsilon_{33}^{T}}E_{3} \end{array}\right.$$

(A6) $$\left\{ \begin{array}{l} S_{1}=s_{11}^{E}T_{1}+d_{31}E_{3}\\ D_{3}=d_{31}T_{1}+\varepsilon_{33}^{T}E_{3} \end{array}\right.$$

For the typical PZT-5H material employed in the sample harvesters:

(A7) $$\begin{array}{l} \left[s^{E}\right]=\left[\begin{array}{cccccc} 16.5 & -4.78 & -8.45 & 0 & 0 & 0\\ -4.78 & 16.5 & -8.45 & 0 & 0 & 0\\ -8.45 & -8.45 & 20.7 & 0 & 0 & 0\\ 0 & 0 & 0 & 43.5 & 0 & 0\\ 0 & 0 & 0 & 0 & 43.5 & 0\\ 0 & 0 & 0 & 0 & 0 & 42.6 \end{array}\right]\times 10^{-12}m^{2}/N\\ \left[d\right]=\left[\begin{array}{cccccc} 0 & 0 & 0 & 0 & 741 & 0\\ 0 & 0 & 0 & 741 & 0 & 0\\ -274 & -274 & 593 & 0 & 0 & 0 \end{array}\right]\times 10^{-12}C/N\\ \left[\varepsilon^{T}\right]=\left[\begin{array}{ccc} 3130 & 0 & 0\\ 0 & 3130 & 0\\ 0 & 0 & 3400 \end{array}\right]\times 8.85\times 10^{-12}F/m \end{array}$$

And the one-dimensional material parameters for plane strain problem can be obtained as:

(A8) $$\left\{ \begin{array}{l} \bar{s_{11}^{E}}=s_{11}^{E}-\frac{s_{12}^{E}s_{12}^{E}}{s_{11}^{E}}=15.12\times 10^{-12}m^{2}/N\\ \bar{d_{31}}=d_{31}-\frac{d_{31}s_{12}^{E}}{s_{11}^{E}}=-353.38\times 10^{-12}C/N\\ \bar{\varepsilon_{33}^{T}}=\varepsilon_{33}^{T}-\frac{{d_{31}}^{2}}{s_{11}^{E}}=2886\times 8.85\times 10^{-12}F/N \end{array}\right.$$

That for the plane stress problem can be obtained as:

(A9) $$\left\{ \begin{array}{l} s_{11}^{E}=16.5\times 10^{-12}m^{2}/N\\ d_{31}=-274\times 10^{-12}C/N\\ \varepsilon_{33}^{T}=3400\times 8.85\times 10^{-12}F/N \end{array}\right.$$

In the main text, the one-dimensional material parameters for the plane stress problem, $s_{11}^{E}$, $d_{31}$, and $\varepsilon_{33}^{T}$, are employed, and the values of these parameters are listed in Table 2.
